# Assembly and analysis of the complete mitochondrial genome of *Forsythia suspensa* (Thunb.) Vahl

**DOI:** 10.1186/s12864-023-09821-4

**Published:** 2023-11-23

**Authors:** Yun Song, Xiaorong Du, Aoxuan Li, Amei Fan, Longjiao He, Zhe Sun, Yanbing Niu, Yonggang Qiao

**Affiliations:** College of Life Sciences, Shanxi Agricultural University, Taigu, Shanxi 030801 China

**Keywords:** *Forsythia suspensa* (Thunb.) Vahl, Mitochondrial genome, Bioinformatics analysis, Phylogenetic evolution

## Abstract

**Background:**

*Forsythia suspensa* (Thunb.) Vahl is a valuable ornamental and medicinal plant. Although the nuclear and chloroplast genomes of *F. suspensa* have been published, its complete mitochondrial genome sequence has yet to be reported. In this study, the genomic DNA of *F. suspensa* yellowish leaf material was extracted, sequenced by using a mixture of Illumina Novaseq6000 short reads and Oxford Nanopore PromethION long reads, and the sequencing data were assembled and annotated.

**Result:**

The *F. suspensa* mitochondrial genome was obtained in the length of 535,692 bp with a circular structure, and the GC content was 44.90%. The genome contains 60 genes, including 36 protein-coding genes, 21 tRNA genes, and three rRNA genes. We further analyzed RNA editing of the protein-coding genes, relative synonymous codon usage, and sequence repeats based on the genomic data. There were 25 homologous sequences between *F. suspensa* mitochondria and chloroplast genome, which involved the transfer of 8 mitochondrial genes, and 9473 homologous sequences between mitochondrial and nuclear genomes. Analysis of the nucleic acid substitution rate, nucleic acid diversity, and collinearity of protein-coding genes of the *F. suspensa* mitochondrial genome revealed that the majority of genes may have undergone purifying selection, exhibiting a slower rate of evolution and a relatively conserved structure. Analysis of the phylogenetic relationships among different species revealed that *F. suspensa* was most closely related to *Olea europaea* subsp. Europaea.

**Conclusion:**

In this study, we sequenced, assembled, and annotated a high-quality *F. suspensa* mitochondrial genome. The results of this study will enrich the mitochondrial genome data of *Forsythia*, lay a foundation for the phylogenetic development of *Forsythia*, and promote the evolutionary analysis of Oleaceae species.

**Supplementary Information:**

The online version contains supplementary material available at 10.1186/s12864-023-09821-4.

## Background

*Forsythia suspensa* (Thunb.) Vahl, commonly known as weeping forsythia, belongs to the Oleaceae family. *F. suspensa* is a deciduous shrub, which is widely distributed in Shanxi, Hebei, Henan, Shaanxi and other provinces of China [1]. The dried fruit of *F. suspensa* (Lian Qiao) is a traditional Chinese medicine (TCM), which is found to exhibit anti-inflammatory, anti-bacterial, anti-viral, anti-oxidative, and immunoregulatory activities [2]. A wide variety of compounds can be isolated from *F. suspensa*, including phenylethanol glycosides, lignanoids, flavonoids, terpenoids, volatile oils, and others. The forsythin and forsythiaside are the main components of lignanoids and phenylethanol glycosides, respectively. They are considered the primary pharmacological compounds in *F. suspensa* [3]. *F. suspensa* is characterized by self-incompatibility and heterostylous floral polymorphisms, including long-styled and short-styled morphologies. Self-pollination among flowers of homomorphic-style plants results in a lower seed-bearing rate than heteromorphic-style plants. Due to distribution patterns and insufficient pollination, natural heterostylous populations of *F. suspensa* tend to experience low seed yield [4]. Recently, Lianhuaqingwen capsules, a Chinese patent medicine with *F. suspensa* as the main raw material, can effectively alleviate the symptoms of COVID-19 [5], so the demand for *F. suspensa* has greatly increased. Because of the ecological, ornamental, and medicinal significance of *F. suspensa*, it is of great significance to study *F. suspensa* deeply.

Mitochondria are the site of energy production in aerobic organisms, including animals and plants. It plays a key role in electron transfer and energy metabolism through oxidative phosphorylation, and provides energy guarantee for various physiological activities of cells [6]. In addition, mitochondria play a role in regulating cellular growth and cycling [7]. Mitochondria originated from the ancestors of prokaryotic endosymbionts, and contain their own genetic materials and systems. But the mitochondrial genome is small and also under the control of the nuclear genome, so mitochondria are considered semi-autonomous organelles [8]. In the early 1860s, Nass et al. [9] first discovered the existence of genetic materials in mitochondria. Subsequently, all the materials required for mitochondrial replication, transcription, and protein translation were discovered, revealing that mitochondria possess a relatively independent genetic expression system [10]. The structure of the mitochondrial genomes varies widely among different phyla of animals and plants. For example, in angiosperms, the mitochondrial genome tends to range in size between 200 and 700 Kb, with some as large as 11 Mb [11]. The mitochondrial genome is rich in repetitive sequences. Due to a large number of exogenous DNA migration and frequent recombination of repeat sequences, its structure has undergone complex changes during evolution [12]. As an independent source of genetic information, the mitochondrial genome is of great significance for studies of species identification, development, and evolution [13].

To date, mitochondrial genomes have been utilized to study the evolutionary history and biochemical processes of various plants. For example, examination of the mitochondrial genome of *Cucumis hystrix* revealed its evolutionary history and patterns of parental inheritance [14]. In *Cyperus esculentus*, as a model system for studying oil accumulation in non-seed tissues, the publication of mitochondrial data lays the foundation for the study of basic biological mechanisms [15]. Although the nuclear and chloroplast genomes of *F. suspensa* have been published, its mitochondrial genome has yet to be reported. Here, we sequenced and assembled the *F. suspensa* mitochondrial genome. Furthermore, we analyzed its structural characteristics, compared its homology with the organellar and nuclear genomes, and evaluated its phylogenetic history and relationships. The *F. suspensa* mitochondrial genome will be indispensable for studies on the evolutionary history and new variety breeding of this valuable ornamental and medicinal species.

## Results

### Characterization and annotation of *F. suspensa* mitochondrial genome

**Fig. 1:**
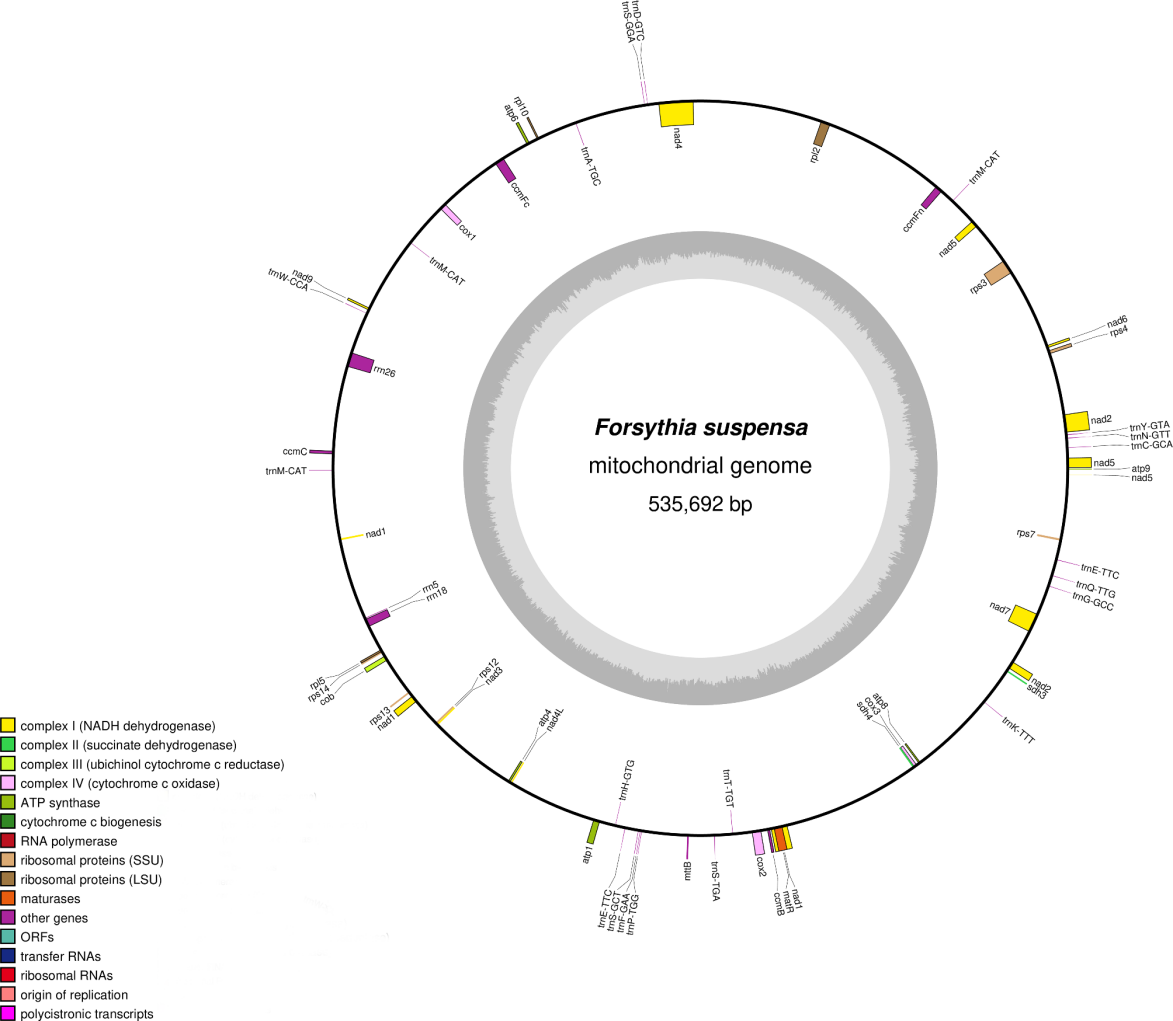
*F. suspensa* mitochondrial genome map

The mitochondrial genome map of *F. suspensa* is shown in Fig. 1. It is a circular structure with a total length of 535,692 bp and GC content of 44.90%. A total of 60 genes were annotated in the genome, including 36 protein-coding genes (PCGs) containing 3102 ORFs, 21 tRNA genes, and three rRNA genes. The predicted genes of *F. suspensa* mitochondrial genome are shown in Table 1. The 36 protein-coding genes are divided into ten categories: ATP synthases (5), Cytochrome c biogenesis (4), Ubiquinol cytochrome c reductase (1), Cytochrome c oxidase (3), Maturases (1), Transport membrane protein (1), NADH dehydrogenases (9), Ribosomal proteins (LSU) (4), Ribosomal proteins (SSU) (6), and Succinate dehydrogenase (2). Among these genes, *nad1*, *nad2*, *nad5*, and *nad7* each contains 4 introns; *nad4* contains 3 introns; and *ccmFc*, *cox2*, and *rps3* each contains one intron. Among the 21 tRNA genes, *trnA-TGC*, *trnE-TTC*, and *trnT-TGT* each contains one intron, and *trnM-CAT* has three copies.

**Table 1:** Classification of genes in the *F. suspensa* mitochondrial genome

Category	Gene name	Length (bp)	Start codon	Stop codon	Amino acid
ATP synthase	*atp1*	1527	ATG	TGA	509
	*atp4*	579	ATG	TAG	193
	*atp6*	720	ATG	CAA(TAA)	240
	*atp8*	480	ATG	TAA	160
	*atp9*	225	TTG	CGA(TGA)	75
Cytochrome c biogenesis	*ccmB*	621	ATG	TGA	207
	*ccmC*	753	ATG	TGA	251
	ccmFc*	1293	ATG	TGA	431
	*ccmFn*	1734	ATG	TGA	578
Ubiquinol cytochrome c reductase	*cob*	1170	ATG	TAA	390
Cytochrome c oxidase	*cox1*	1584	ATG	TAA	528
	cox2*	783	ATG	TAA	261
	*cox3*	798	ATG	TGA	266
Maturases	*matR*	1968	ATG	TAG	656
Transport membrane protein	*mttB*	348	ATG	TAG	116
NADH dehydrogenase	nad1****	978	ATG	TAA	326
	nad2****	1467	ATG	TAA	489
	*nad3*	357	ATG	TAA	119
	nad4***	1479	ATG	TGA	493
	*nad4L*	303	ACG (ATG)	TAA	101
	nad5****	2013	ATG	TAA	671
	*nad6*	618	ATG	TAA	206
	nad7****	1185	ATG	TAG	395
	*nad9*	573	ATG	TAA	191
Ribosomal proteins (LSU)	*rpl10*	492	ATG	TAA	164
	rpl2*	993	ATG	TAA	331
	*rpl5*	549	ATG	TAA	183
	#*rpl16*				
Ribosomal proteins (SSU)	*rps12*	378	ATG	TGA	126
	*rps13*	351	ATG	TGA	117
	*rps14*	303	ATG	TAG	101
	rps3*	1686	ATG	TAA	562
	*rps4*	819	ATG	TAA	273
	*rps7*	447	ATG	TAA	149
Succinate dehydrogenase	*sdh3*	312	ATG	TGA	104
	*sdh4*	387	ATG	CGA (TGA)	129
Ribosomal RNAs	*rrn18*	1935			
	*rrn26*	3405			
	*rrn5*	121			
Transfer RNAs	trnA-TGC*	65			
	*trnC-GCA*	71			
	*trnD-GTC*	74			
	trnE-TTC*	72			
	*trnE-TTC*	69			
	*trnF-GAA*	74			
	*trnG-GCC*	72			
	*trnH-GTG*	74			
	*trnK-TTT*	73			
	*trnM-CAT* (3)	77,74,73			
	*trnN-GTT*	72			
	*trnP-TGG*	75			
	*trnQ-TTG*	72			
	*trnS-GCT*	88			
	*trnS-GGA*	87			
	*trnS-TGA*	87			
	trnT-TGT*	72			
	*trnW-CCA*	74			
	*trnY-GTA*	83			

Notes: *, intron number; #, Pseudogene; Gene (3), Number of copies of multi-copy genes

### RNA editing analysis

**Fig. 2:**
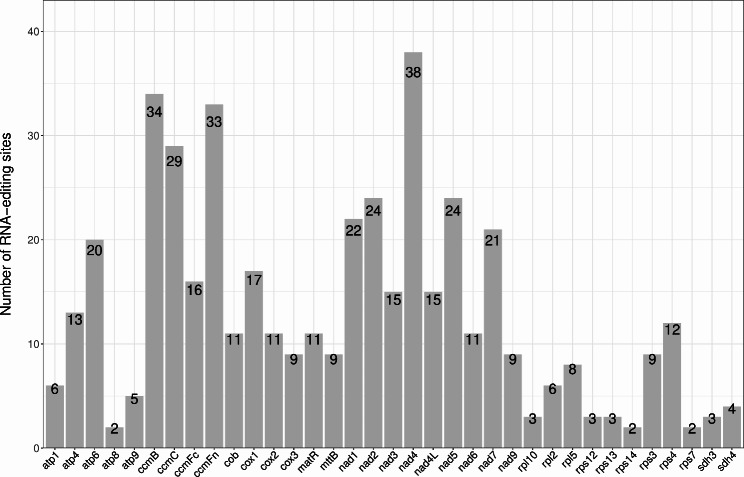
RNA editing sites in the *F. suspensa* mitochondrial genome

There are 460 predicted RNA editing sites in the 36 protein-coding genes in the *F. suspensa* mitochondrial genome. As shown in Fig. 2 and 38 RNA editing sites are in *nad4*, 34 are in *ccmB*, 33 are in *ccmFn*, and two each are in *atp8*, *rps14*, and *rps7*. RNA editing is known to alter the hydrophilic and hydrophobic properties of the encoded amino acid residues. Overall, we found that the properties of 44.78% of amino acid residues are unchanged, 47.17% of amino acid residues are changed from hydrophilic to hydrophobic, and 7.61% of amino acid residues are changed from hydrophobic to hydrophilic, as shown in Table 2. All identified RNA-editing sites are of the pyrimidine-thymine (C-T) type. Among these, 136 (29.57%) of the editing sites are located on the first base of the triplet codon, and 304 (66.09%) of the editing sites are located on the second base of the triplet codon. In addition, 20 (4.35%) editing sites are located at the first and second position of the triplet codon, resulting in a change from proline (CCC/CCT) to phenylalanine (TTC/TTT). Furthermore, RNA editing may lead to the premature termination of the protein-coding genes *atp6* and *atp9* [16]. We found that after editing, most amino acid residues are changed to leucine (L) (46.52%) and phenylalanine (F) (22.39%).

**Table 2:** Analysis of RNA editing sites and types in the *F. suspensa* mitochondrial genome

Type of edit	RNA editing	Number	Percentage
Hydrophilic-hydrophilic	CAC (H) = > TAC (Y)	6	
	CAT (H) = > TAT (Y)	15	
	CGC (R) = > TGC (C)	7	
	CGT (R) = > TGT (C)	29	
	total	57	12.39%
Hydrophilic-hydrophobic	ACA (T) = > ATA (I)	3	
	ACC (T) = > ATC (I)	1	
	ACG (T) = > ATG (M)	3	
	ACT (T) = > ATT (I)	3	
	CGG (R) = > TGG (W)	28	
	TCA (S) = > TTA (L)	72	
	TCC (S) = > TTC (F)	31	
	TCG (S) = > TTG (L)	38	
	TCT (S) = > TTT (F)	38	
	total	217	47.17%
Hydrophilic-stop	CAA (Q) = > TAA (X)	1	
	CGA (R) = > TGA (X)	1	
	total	2	0.43%
Hydrophobic-hydrophilic	CCA (P) = > TCA (S)	6	
	CCC (P) = > TCC (S)	6	
	CCG (P) = > TCG (S)	3	
	CCT (P) = > TCT (S)	20	
	total	35	7.61%
Hydrophobic-hydrophobic	CCA (P) = > CTA (L)	44	
	CCC (P) = > CTC (L)	9	
	CCC (P) = > TTC (F)	6	
	CCG (P) = > CTG (L)	31	
	CCT (P) = > CTT (L)	20	
	CCT (P) = > TTT (F)	14	
	CTC (L) = > TTC (F)	4	
	CTT (L) = > TTT (F)	10	
	GCC (A) = > GTC (V)	2	
	GCG (A) = > GTG (V)	7	
	GCT (A) = > GTT (V)	2	
	total	149	32.39%

Note: Type of edit, type of hydrophilic and hydrophobic change; RNA editing, RNA editing type; Number, incidence of RNA editing type; Percentage, the percentage of each change among all changes

### Relative Synonymous Codon Usage (RSCU) analysis

**Fig. 3:**
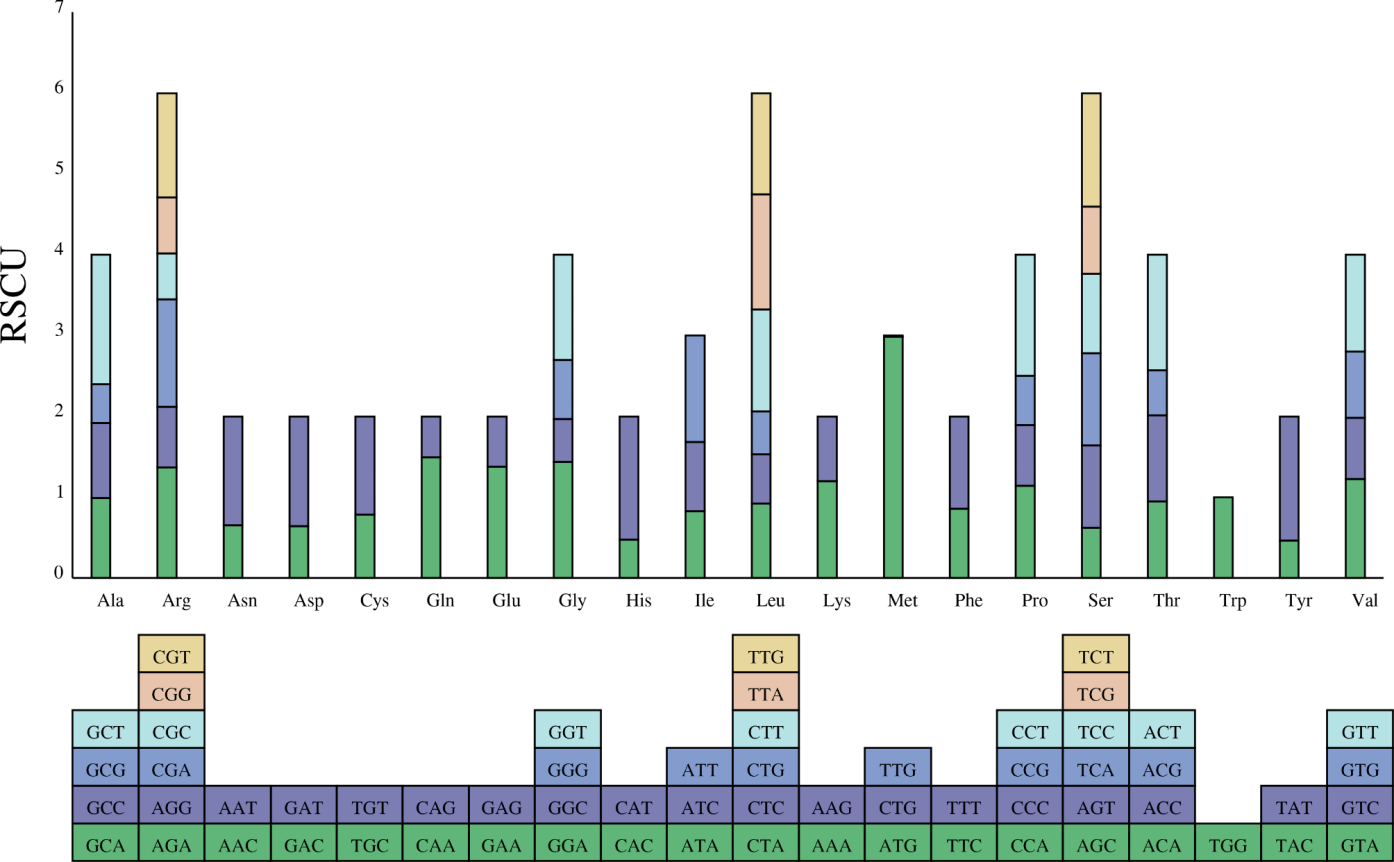
Analysis of relative synonymous codon usage (RSCU) in the *F. suspensa* mitochondrial genome Note: The following blocks represent all codons encoding each amino acid, and the height of the upper column represents the sum of RSCU values of all codons

The analysis of the relative synonymous codon usage of *F. suspensa* is shown in Fig. 3. In the *F. suspensa* mitochondrial genome, the RSCU value of UGG (Trp) is equal to one, and its actual usage frequency equals its theoretical frequency. Thirty-one codons are identified to have an RSCU > 1. The most frequently used codon, AUG (Met), has an RSCU value of 2.98. Codons GCU (Ala), UAU (Tyr), and CAU (His) are used less frequently, with CUG (Met) and UUG (Met) exhibiting the least frequent use. In addition, codons ending in A/T account for 90.32% (28) of the total, indicating that the codons in the *F. suspensa* mitochondrial genome are A/T biased.

### Repeat sequence analysis

**Fig. 4:**
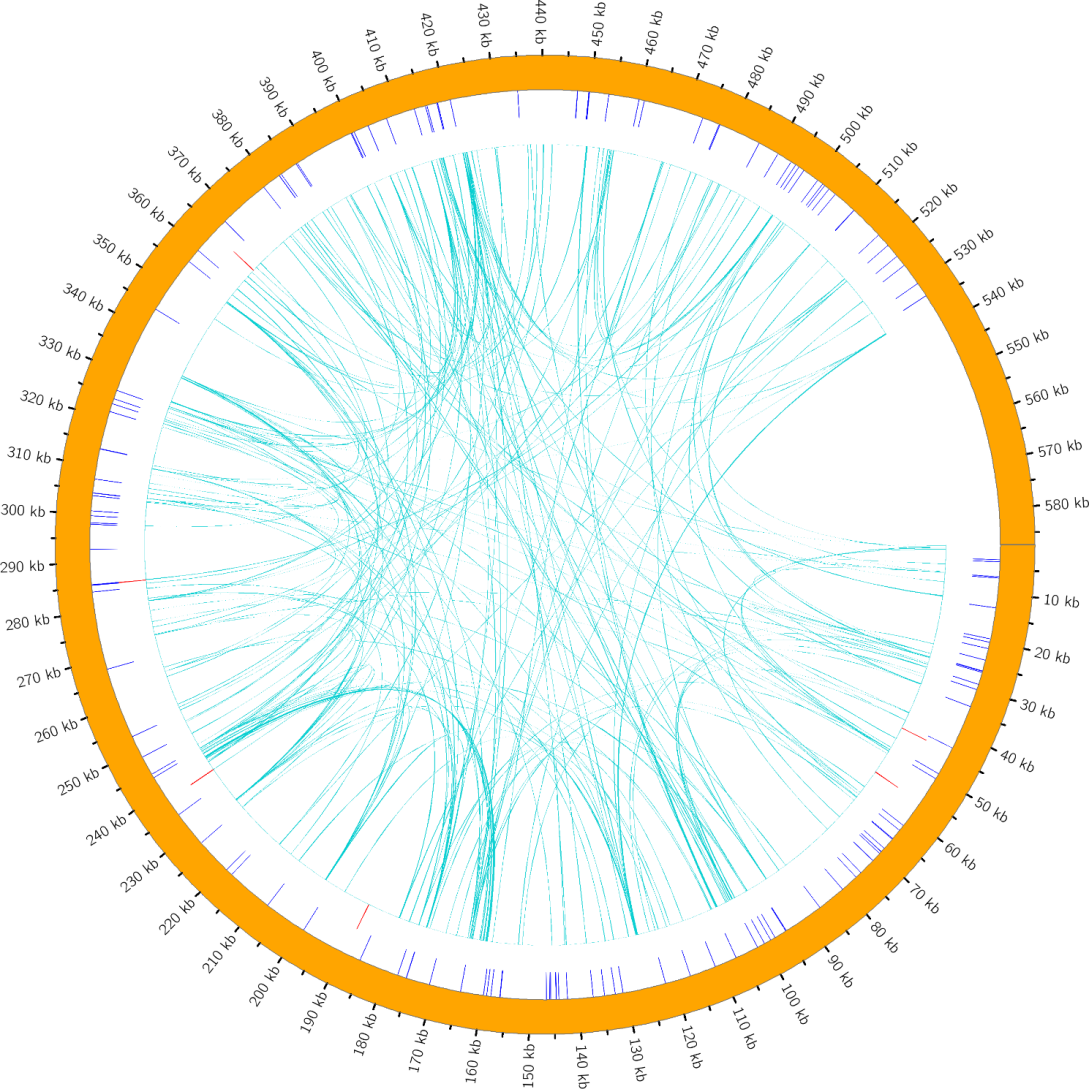
Distribution map of repeat sequences in the *F. suspensa* mitochondrial genome Note: The outermost circle represents simple repeat sequences, followed by tandem repeat sequences, and the innermost line represents scattered repeat sequences

We identified 364 repeat sequences in the *F. suspensa* mitochondrial genome, including 137 simple sequence repeats (SSRs), 9 tandem sequence repeats (TSRs), and 218 dispersed sequence repeats (DSRs), as shown in Fig. 4. Among the SSRs, monomers, dimers, and tetramers account for 30.66% (42), 24.09% (33), and 27.01% (37), respectively, with hexamers accounting for only 0.73% (1). Adenine (A) mononucleotide repeats account for 50% (21) of the monomeric SSRs, and AG/TA repeats account for 24.24% (8) of the dimeric SSRs (Supplementary Fig. 1). With the exception of ATATTGATG (70%), the nine TSRs exhibit a matching degree of over 82%, and range from 2 to 36 bp in length (Supplementary Table 1). DSRs include forward repeats, palindromic repeats, reverse repeats, and complement repeats. Only 52.29% (114) of the palindromic sequences and 47.71% (104) of the forward repeat sequences are identified in the *F. suspensa* mitochondrial genome.

### Homologous sequence analysis of organellar and nuclear genomes

**Fig. 5:**
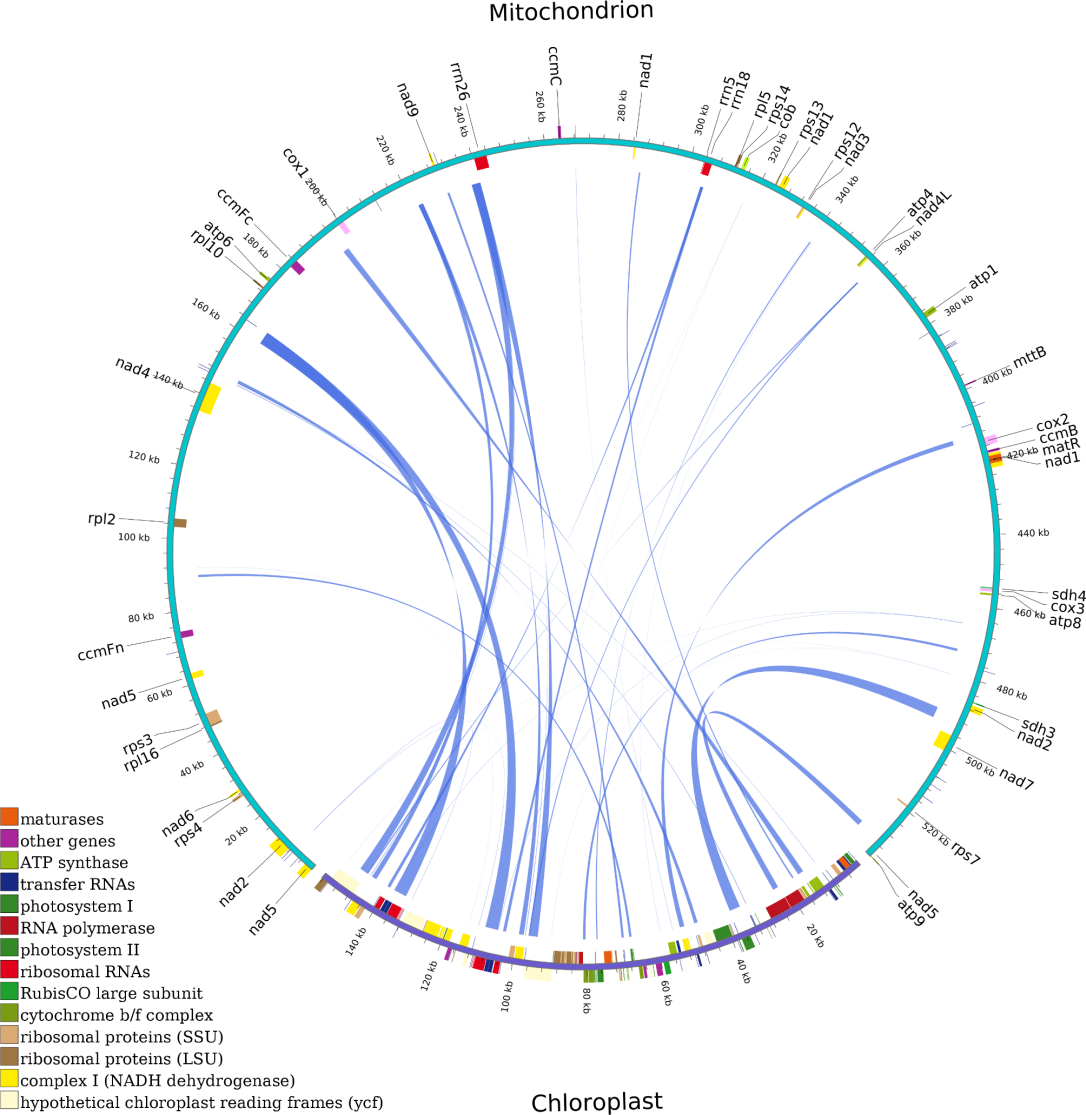
Schematic representation of the homologous fragments from the *F. suspensa* chloroplast and mitochondrial genomes

Homology comparison between the *F. suspensa* mitochondrial and chloroplast genomes as shown in Fig. 5. We identified 25 homologous sequences with a total length of 21,037 bp, accounting for approximately 3.93% of the total mitochondrial genome. The longest homologous fragment is 3,704 bp and the shortest is 30 bp. As shown in Table 3, the *F. suspensa* chloroplast genome contains several genes which are completely integrated and 100% matched to the mitochondrial genome, including *trnA-UGC*, *psbC*, *psbD*, *ycf15*, *atpH*, *trnN-GUU*, *trnM-CAU*, *trnD-GUC*, *psbJ*, *psbL*, *psbF*, *trnS-GGA*, *petG*, *trnW-CCA*, *trnP-UGG*, *ndhK*, and *trnS-GCU.* All chloroplast genes undergoing transfer correspond to the mitochondrial genome as *trnA-TGC*, *trnS-GGA*, *trnW-CCA*, *trnD-GTC*, *trnN-GTT*, *trnM-CAT*, *trnS-GGA*, and partial *rrn18*.

**Table 3:** Analysis of homologous fragments of the *F.suspensa* chloroplast and mitochondrial genomes

No.	Length(bp)	Number of mism-atches	Num-ber of gap open-ings	Cp start	Cp end	Mt start	Mt end	Genes (cp.)	Genes (mt)
1	3704	0	1	105,768	109,471	163,780	160,078	*trnI-GAU* (partical:22.57%); *trnA-UGC*; *rrn23* (partical:84.23%)	*trnA-TGC*
				134,093	137,796	160,078	163,780		
2	3179	0	2	34,479	37,657	490,717	493,893	*psbD*; *psbC*	
3	2431	3	0	147,015	149,445	240,258	237,828	*ycf15*; *ycf2* (partical:29.92%)	
				94,119	96,549	237,828	240,258		
4	1730	26	12	13,523	15,249	195,898	197,601	*atpF* (partical:40.56%); *atpH*	
5	1662	25	9	22,086	23,738	530,312	531,943	*rpoC1* (partical:58.58%)	
6	1406	0	0	143,959	145,364	222,781	221,376	*ndhB* (partical:63.56%)	
				98,200	99,605	221,376	222,781		
7	735	11	4	80,291	81,024	472,900	473,621	*petD* (partical:46.49%)	
8	1005	108	25	47,478	48,474	146,737	147,705	*trnS-GGA*; *rps4* (partical:80.53%)	*trnS-GGA*
9	1365	187	46	51,487	52,821	412,184	413,485	*ndhJ* (partical:71.70%); *ndhK*; *ndhC* (partical:42.70%)	
10	433	11	2	16,887	17,318	286,972	286,547	*rps2* (partical:60.76%)	
11	676	55	20	66,987	67,651	89,732	89,092	*psbJ*; *psbL*; *psbF*; *psbE* (partical:45.24%)	
12	349	1	0	76,781	77,129	340,188	340,536	*psbB* (partical:22.86%)	
13	389	14	5	96,438	96,810	358,600	358,212	*ycf15* (partical:35.86%)	
				146,754	147,126	358,212	358,600		
14	674	71	25	69,221	69,878	231,054	230,418	*petG*; *trnW-CCA*; *trnP-UGG*	*trnW-CCA*
15	885	186	37	103,403	104,266	305,615	304,757	*rrn16* (partical:57.99%)	*rrn18* (partical:44.39%)
				139,298	140,161	304,757	305,615		
16	149	2	0	145,776	145,924	240,392	240,244	*ndhB* (partical:6.74%)	
				97,640	97,788	240,244	240,392		
17	163	15	2	32,451	32,611	146,276	146,114	*trnD-GUC*	*trnD-GTC*
18	90	2	1	105,634	105,723	465,356	465,269	*trnI-GAU* (partical:8.83%)	
				137,841	137,930	465,269	465,356		
19	85	3	1	111,380	111,463	6992	6908	*trnN-GUU*	*trnN-GTT*
				132,101	132,184	6908	6992		
20	80	5	0	54,948	55,027	268,269	268,348	*trnM-CAU*	*trnM-CAT*
21	47	0	0	58,618	58,664	340,860	340,814	*rbcL* (partical:3.28%)	
22	32	0	0	114,267	114,298	91,814	91,845	*ndhF* (partical:1.44%)	
23	90	16	3	9316	9404	146,933	146,846	*trnS-GCU*	*trnS-GGA*
24	31	0	0	90,116	90,146	480,491	480,461	*ycf2* (partical:0.45%)	
				153,418	153,448	480,461	480,491		
25	30	0	0	103,391	103,420	317,854	317,825	*rrn16* (partical:2.01%)	
				140,144	140,173	317,825	317,854		

Homology comparison between the *F. suspensa* mitochondrial and nuclear genomes identified 9,473 homologous fragments spanning 14 chromosomes. Among them, the longest sequence is located on chromosome 3, with a total length of 10,677 bp and accounting for 2% of the mitochondrial genome. Fifty-six homologous fragments are greater than 1,000 bp in length, and are primarily located on chromosome 13 (Supplementary Fig. 2).

### Ka/Ks and Pi analyses

**Fig. 6:**
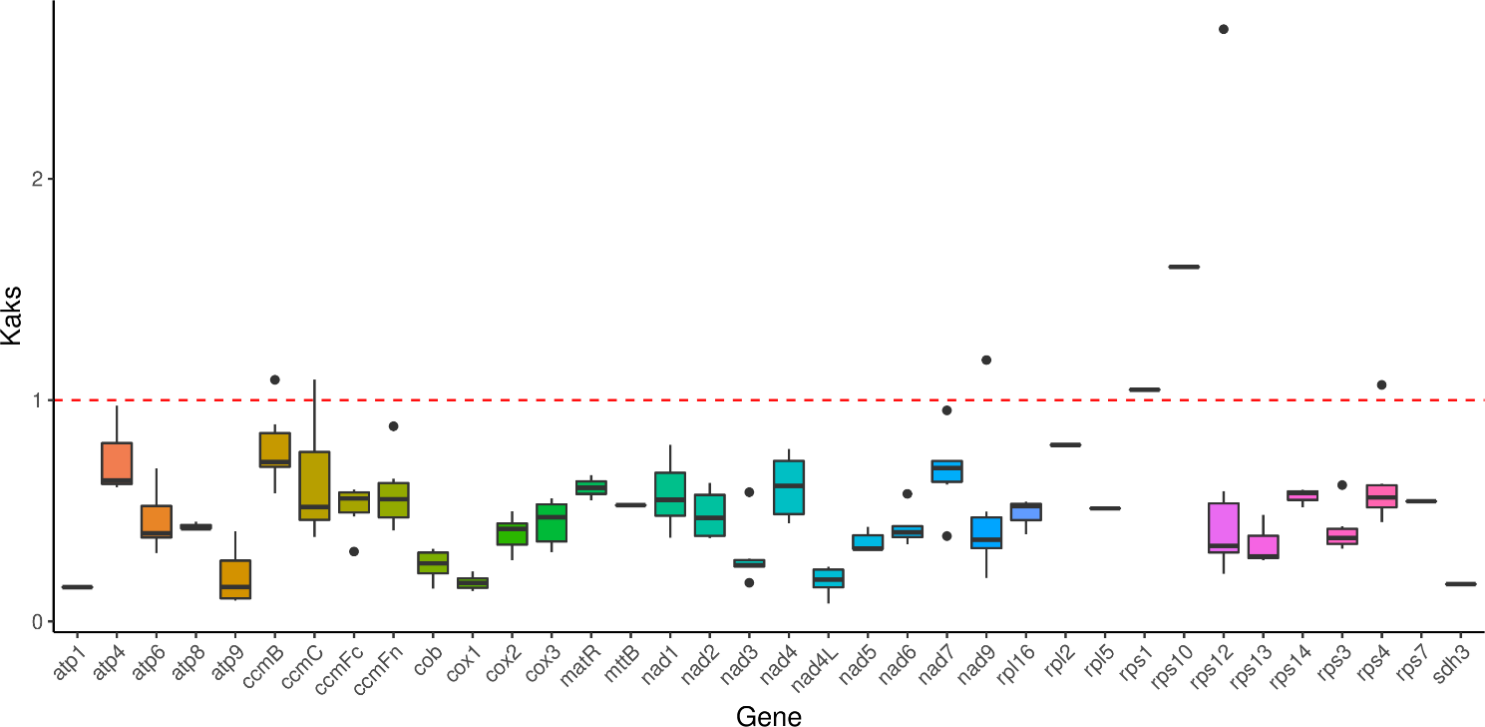
Boxplots of the Ka/Ks ratio of *F. suspensa* and four other plant species

A ratio of non-synonymous mutation rate (Ka) to synonymous mutation rate (Ks) greater than 1, less than 1, and equal to 1 indicates positive selection, purifying selection, and neutral selection, respectively [17]. Fig. 6 shows the Ka/Ks ratios obtained by comparing the mitochondrial genomes of *F. suspensa*, *Olea europaea* subsp. Europaea (*O. europaea*), *Nicotiana tabacum* (*N. tabacum*), *Zea mays* subsp. Mays (*Z. mays*), and *Glycine max* (*G. max*). Both *rps1* and *rps10* exhibit a Ka/Ks ratio > 1, indicating that they might be affected by positive selection. The genes *atp1*, *atp9*, *cob*, *cox1*, *nad4L*, and *sdh3* exhibit Ka/Ks ratios < 1, indicating that they might be affected by purifying selection and are relatively conserved during evolution. Comparison of *F. suspensa* to *O. europaea* (both in the Oleaceae family) revealed that *atp4* and *sdh3* are subject to positive selection.

**Fig. 7:**
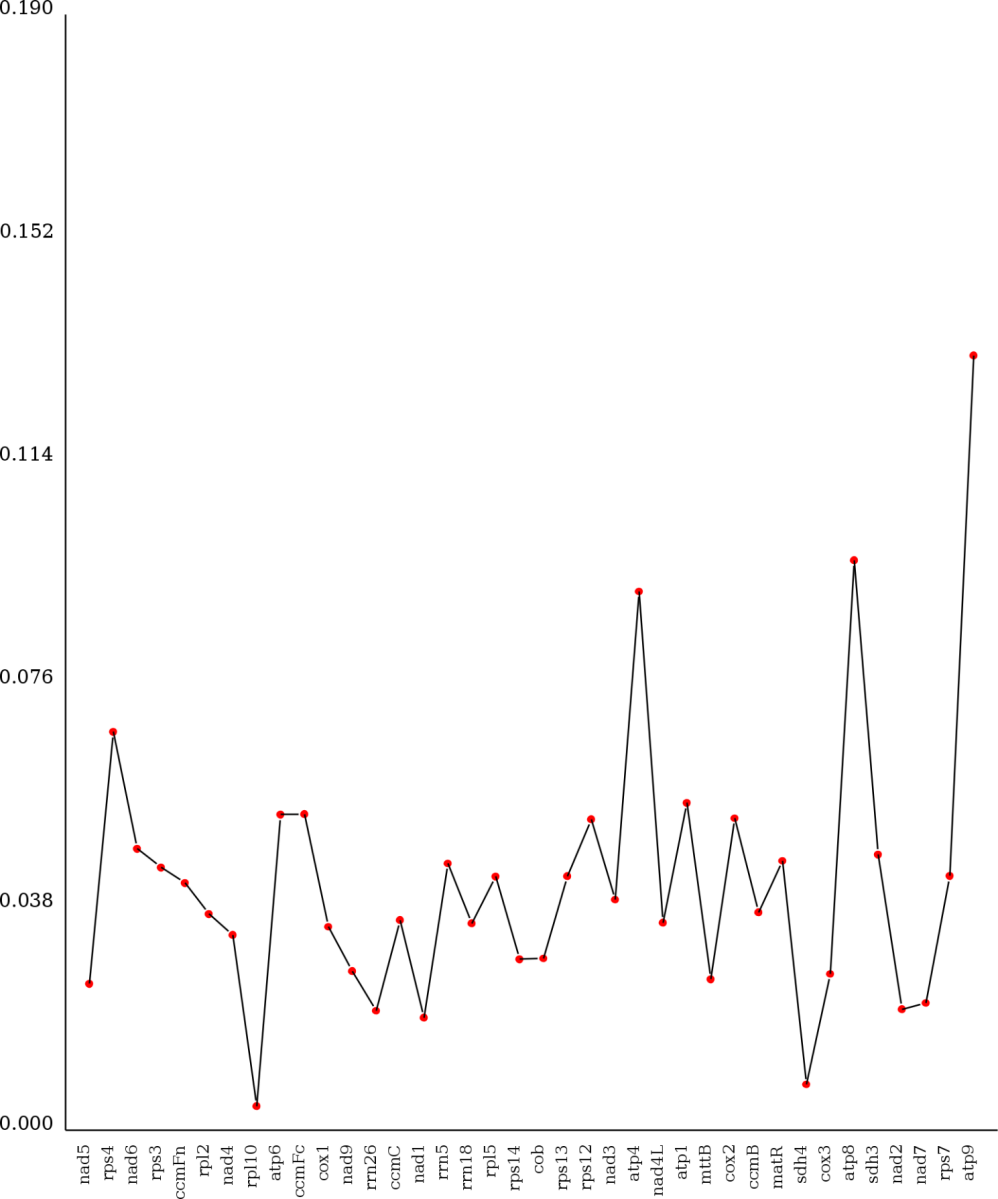
Line plots of Pi values of *F. suspensa* and four other plant species Note: The abscissa represents the gene name, and the ordinate represents the Pi value

Nucleic acid diversity (Pi) can reveal the magnitude of variation in nucleic acid sequences of different species, and regions with higher variability can provide potential molecular markers for population genetics studies [18]. As shown in Fig. 7, a global comparison of the homologous gene sequences of *F. suspensa*, *O. europaea*, *N. tabacum*, *Z. mays*, and *G. max* found that *atp9* exhibits the highest degree of variation, followed by *atp8*, *atp4*, and *rps4.* Both *rpl10* and *sdh4* exhibit a relatively low degree of variation, and thus are more conserved.

### Structural comparative analysis and collinear analysis

The *F. suspensa* mitochondrial genome exhibits distinct gene distributions on the sense and antisense strands. Comparison of the structure of the *F. suspensa* mitochondrial genome with those of *O. europaea*, *N. tabacum*, *Z. mays*, and *G. max* revealed that *O. europaea* and *F. suspensa* share the greatest number of homologous sequences and a higher degree of homology. By contrast, the *F. suspensa* mitochondrial genome shares a smaller number of homologous sequences with *N. tabacum*, *Z. mays*, and *G. max*. Furthermore, the *F. suspensa* mitochondrial genome is relatively evolutionarily conserved and exhibits no obvious GC skew (Supplementary Fig. 3).

**Fig. 8:**
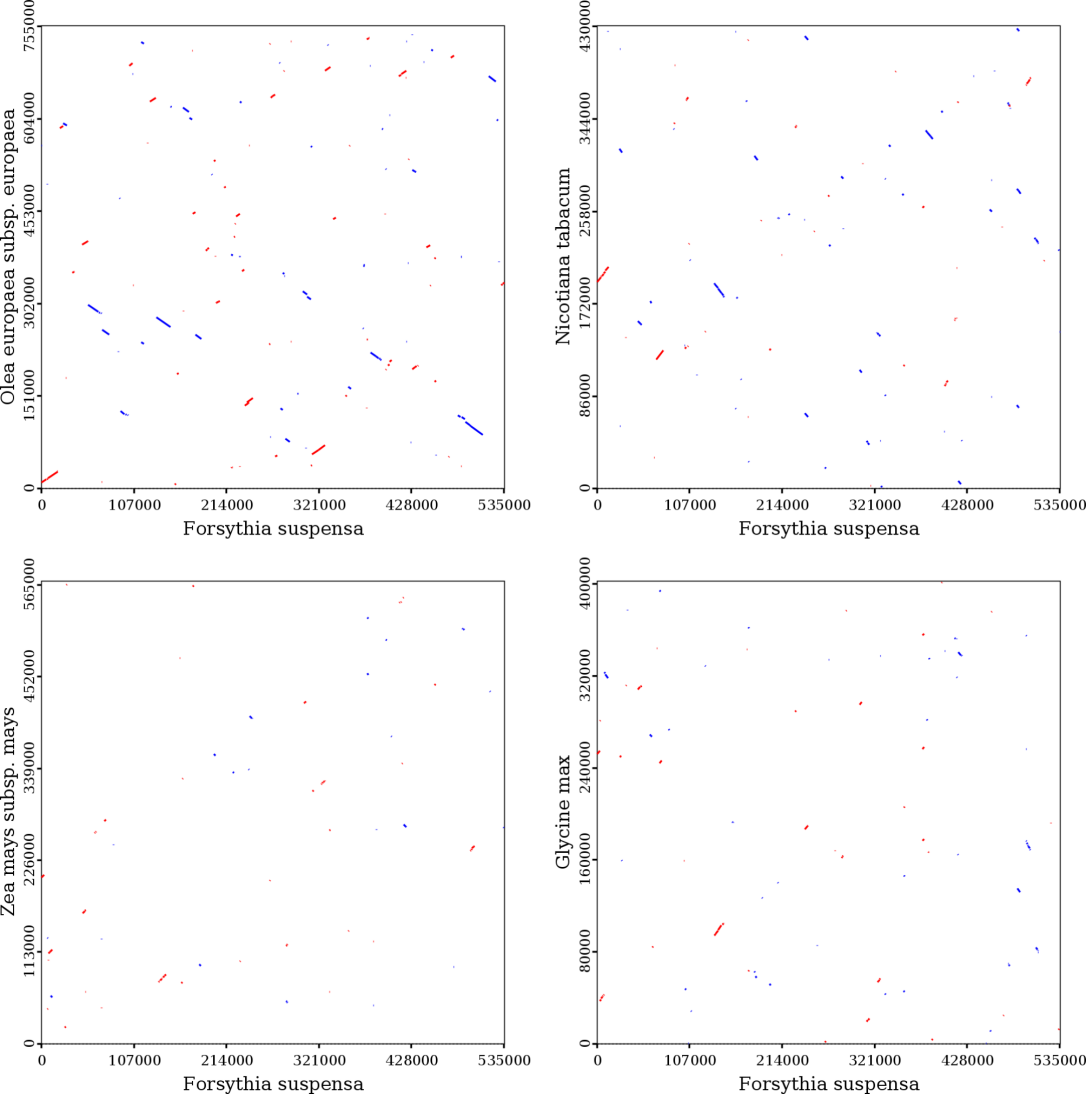
Collinear dot plot analysis of the *F. suspensa* mitochondrial genome Note: In each box, the abscissa represents the assembled sequence, the ordinate indicates other sequences, the red line indicates the forward alignment, and the blue line indicates the reverse complementary alignment

**Fig. 9:**
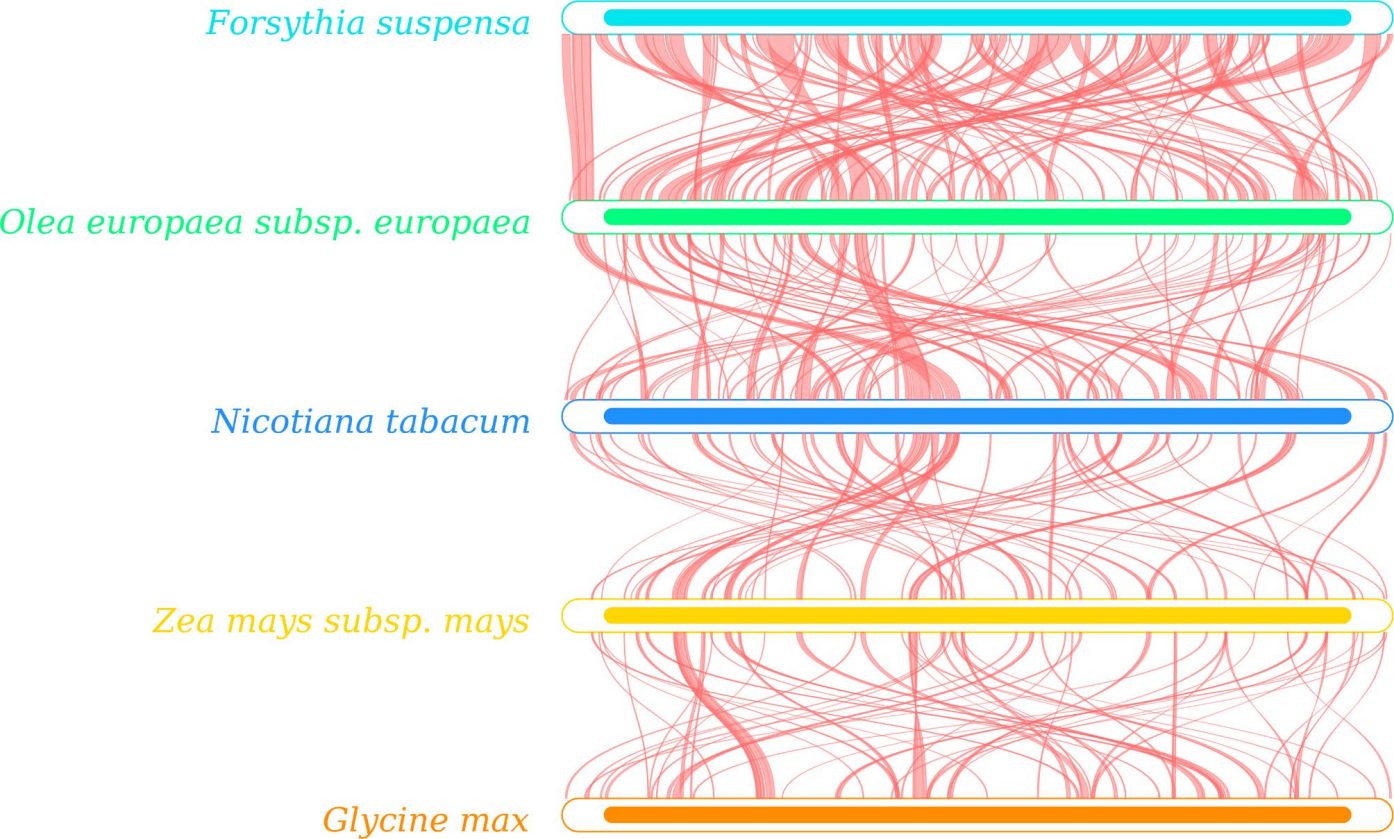
Synteny analysis of the *F. suspensa* mitochondrial genome

The collinearity analysis of *F. suspensa* mitochondrial genome is shown in Fig. 8. There are many collinear blocks between *F. suspensa* and *O. europaea*, most of which are more than 90% homologous. The longest homologous sequence is approximately 13,700 bp in length, with a fully matched sequence between 68 and 312 bp. The longest collinear block in *N. tabacum* is about 8,140 bp, with greater than 93% homology. In addition, two homologous fragments with lengths of 66 and 89 bp are completely matched to the *F. suspensa* mitochondrial genome. There are smaller collinear blocks in *Z. mays* and *G. max*, among which two 77 bp fragments in *Z. mays* are 100% homologous with *F. suspensa*. As shown in Fig. 9, synteny analysis identified many homologous collinear blocks between *F. suspensa* and closely related species. These results indicate that the rearrangement of functional genes in the *F. suspensa* mitochondrial genome maintained synteny, which is also highly conserved in protein-coding.

### Phylogenetic analysis

**Fig. 10:**
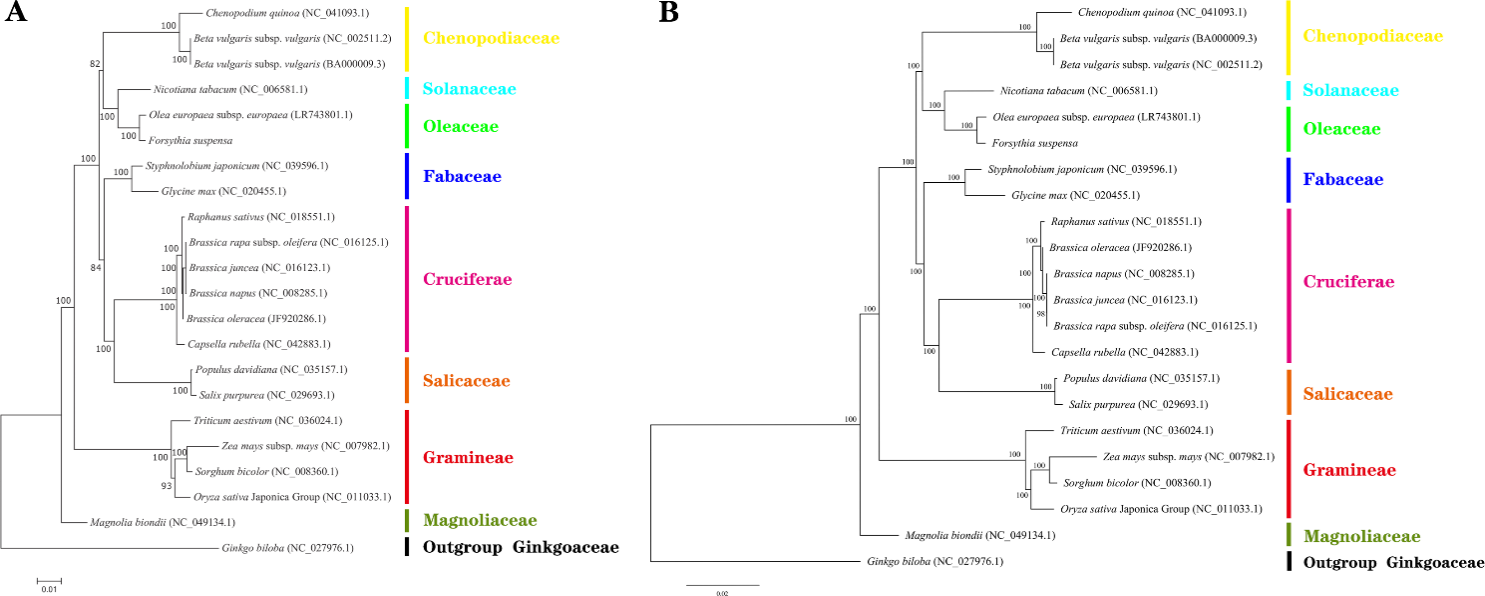
Phylogenetic analysis of the mitochondrial genomes of 22 plant species Note: **A**: Phylogenetic tree constructed using the maximum-likelihood method; **B**: Phylogenetic tree constructed using the Bayesian method

Cluster analysis of the mitochondrial genomes of 22 plant species, including *F. suspensa*, was used to create a phylogenetic tree as shown in Fig. 10. Using *Gingko biloba* (Ginkgoaceae)as the outgroup, all other plant species were clustered into four groups. *F. suspensa* and *O. europaea* are clustered into one category, and the genetic relationship is the closest. They were clustered into the first category with *Beta vulgaris* (both sugar beets and red beets), *Chenopodium quinoa* and *N. tabacum*. Therefore, the clustering results showed that Oleaceae was closely related to Solanaceae and Chenopodiaceae. The species of Cruciferae, Salicaceae and Fabaceae, which are clustered into the second category, have a distant relationship with Oleaceae. The species of Gramineae and Magnoliaceae belong to the third and fourth categories, respectively, and have the farthest genetic relationship with Oleaceae.

## Discussion

The structure of the plant mitochondrial genome is diverse, polymorphic, and complex [13]. The majority of plant mitochondrial genomes are circular, although some are H-shaped, Y-shaped, linear, or both linear and circular [19]. In this study, we used Illumina Novaseq6000 and Oxford Nanopore PromethION to sequence and assemble the mitochondrial genome of *F. suspensa*. We obtained the mitochondrial genome data of *F. suspensa* for the first time, and its structure is circular. The mitochondrial genome structure of *F. suspensa* is the same as that of most plants, such as *Vitex rotundifolia* [20] and *Bupleurum chinense* [21] with circular mitochondrial genomes. This is quite different from the linear structure of japonica rice [22]. Although angiosperm mitochondrial genomes are highly variable in size between, and even within, species, the number of encoded functional genes is relatively conserved [13, 23, 24]. The mitochondrial genome of *Silene conica* can reach 11 Mb, which is the largest known mitochondrial genome among angiosperms. By contrast, the mitochondrial genome of *B. napus* is only 221 Kb. We found that the length of *F. suspensa* mitochondrial genome is 535,692 bp, similar to that of the annual wild rice mitochondrial genome [25]. The number of genes encoded by plant mitochondrial genomes is relatively consistent, including genes encoding complexes I, II, III, IV, and V of the respiratory chain, severl cytochrome c synthetase subunits, as well as tRNA and rRNA [26]. Among the 60 genes encoded in the *F. suspensa* mitochondrial genome, 36 are protein-coding genes, 21 are tRNA genes, and three are rRNA genes, which is almost consistent with studies in other species. In higher plants, parts of the genes are often lost, resulting in the creation of pseudogenes without expression or normal function [27]. The mitochondrial gene annotation of *F. suspensa* contains the pseudogene *rpl16*, which may have lost its function during evolution.

An important source of variation among angiosperm mitochondrial genomes is RNA editing, which can alter the expression patterns of mitochondrial genes. The number of editing sites varies among different species, which is likely related to their evolutionary history [28, 29]. In addition, RNA editing can promote RNA splicing by cutting intron structure splicing, which plays an important role in plant evolutionary adaptation and development [30]. For example, the mitochondrial genome of *O. sativa* contains 491 RNA editing sites, while that of *B. vulgaris* contains 357 RNA editing sites [23]. We found 460 RNA editing sites in the *F. suspensa* mitochondrial genome, it mainly occurs in the first and second positions of codons, accounting for 95.65% of all editing sites. The editing method is cytosine = > thymine (C = > T), which is consistent with the research of Sloan et al. [31]. The remaining 4.35% of RNA editing sites occurred in the top two codons at the same time, which was similarly found in *C. hystrix* [14]. The RNA editing sites occurred in almost all protein-coding genes, indicating that the mitochondrial gene of *F. suspensa* may have an important effect on the function of RNA editing sites.

Codon bias is the combined result of natural selection, species mutation, and genetic drift. The most frequently used codon in the *F. suspensa* mitochondrial genome is the initiation codon AUG, followed by GCU (Ala), UAU (Tyr), and CAU (His), similar to the codon usage in *Quercus acutissima* [32]. Further analysis revealed a preference for A/T bases in the *F. suspensa* mitochondrial genome, Similar analysis of codon bias has been performed in mitochondrial genome data of multiple species [33, 34], providing a lot of information for the evolution of species. Equally, RSCU analysis can provide a new basis for exploring the position of *F. suspensa* in phylogeny.

Sequence repeats in the mitochondria genome result in intermolecular recombination, which is the primary driver of mitochondrial genomic evolution [35]. As a result of their evolutionary history, different species, or different varieties of the same species, contain different repeat sequences [36]. Repeated sequences can range from a few bp to tens of Kb. Long sequence repeats account for the majority of intramolecular or intermolecular recombination in the mitochondrial genome, such as *atp6* in *Arabidopsis thaliana*. SSRs tend to mediate mitochondrial genomic rearrangement. For example, the mitochondrial genome of *Cucurbita pepo* contains a total of 371 Kb of SSRs [37]. We found that the *F. suspensa* mitochondrial genome contains 364 sequence repeats. Simple sequence repeats contain a variety of nucleotide repeats, which can be used as an important molecular marker for the identification of *F. suspensa*. A large number of repetitive sequences in the mitochondrial genome of *F. suspensa* are partially or completely covered in the mitochondrial gene sequence, which may change the function of mitochondrial genes due to intermolecular recombination, which provides a reference for the gene rearrangement event of the mitochondrial genome of *F. suspensa.* Repeat sequence analysis became an important factor in mitochondrial genome evolution. *Agrostis stolonifera* contains frequent repeat sequences, including SSRs, TSRs, DSRs, which make the mitochondrial genome appear multiple loop structure, and the molecular rearrangement may also affect some functions of the species [38].

Intracellular gene transfer among the mitochondrial, chloroplast, and nuclear genomes has become a popular research focus. For example, the plastid-derived gene *rpl32* has been transferred into the nucleus of the subfamily Thalictroideae (Ranunculaceae) [39]. Comparison of the chloroplast and mitochondrial genomes of *F. suspensa* identified numerous transferred fragments and 17 completely transferred chloroplast genes, including *trnA-UGC, psbC, psbD, ycf15, and atpH*, etc. In higher plants, compared to the complex mitochondrial genome, chloroplast genomes are relatively stable, with mangy conserved genes and a conserved arrangement order [40]. For example, the order of gene arrangement in the chloroplast genomes is identical between *O. sativa*, *Z. mays*, and *T. aestivum*, although their mitochondrial genomes are quite different. The mitochondrial genome of *Vitis vinifera* contains 30 fragments from its chloroplast genome, equivalent to 8.8% of its entire mitochondrial genome [41]. In this study, the mitochondrial genome of *F. suspensa* contains 25 fragments from its chloroplast genome, accounting for about 3.93% of the mitochondrial genome. There are also a large number of homologous fragments between the nuclear genome and the mitochondrial genome. By identifying the existence of these homologous fragments, it can be further confirmed that there is a wide range of DNA exchange between the organelle genome and the nuclear genome of *F. suspensa*.

In this study, the mitochondrial genome of *F. suspensa* was compared with the mitochondrial genomes of its related species *O. europaea* and other three common species. It was found that most of the genes in the mitochondrial genome of *F. suspensa* have undergone negative selection. Many of these are highly homologous to genes in the *O. europaea* mitochondrial genome, reaching up to 100% homogeneity. The inconsistency of the collinearity analysis indicated that *F. suspensa* may have undergone gene rearrangement and different degrees of selective evolution. Based on previous comparative studies of the chloroplast and nuclear genomes of the Forsythieae, phylogenetic analysis supports the monophyly of *Forsythia*. Its distribution and evolutionary relationships in Eurasia are also explained [42]. Reconstruction of the phylogeny using organellar and nuclear genomes information from Oleaceae species, but some phylogenetic relationships between the tribes are still difficult to resolve [43]. By using multiple sequence datasets and various phylogenomics approaches to infer the phylogenetic relationships of Oleaceae, introgression events occurred during the diversification of Oleaceae [44]. The evolutionary relationships among multiple tribes of Oleaceae remain poorly defined, in this study, phylogenetic analysis of *F. suspensa* and 21 other species of plants revealed that *F. suspensa* was most closely related to *O. europaea*, both of the family Oleaceae. The evolution of mitochondrial genome is slow and conservative. The acquisition of mitochondrial genome data and phylogenetic analysis of *F. suspensa* provide a prerequisite for the exploration of the evolutionary history of *F. suspensa*, and the data for the taxonomic and evolutionary study of Oleaceae species.

## Conclusions

In this study, we sequenced, assembled, and annotated a high-quality *F. suspensa* mitochondrial genome. The *F. suspensa* mitochondrial genome was obtained in the length of 535,692 bp with a circular structure and a GC content of 44.90%. The genome contains 60 genes, including 36 protein-coding genes, 21 tRNA genes, and three rRNA genes. The *F. suspensa* mitochondrial genome contains 460 predicted RNA editing sites, and exhibits a bias toward A/T bases. Homologous sequence comparison of organellar and nuclear genomes identified chloroplast gene transfer events. Nucleic acid diversity analysis provided a reference for identifying potential molecular markers in *F. suspensa*. The *F. suspensa* mitochondrial genome has undergone gene rearrangement during evolution, and the majority of coding genes have undergone purifying selection, indicating that mitochondrial genes are largely conserved during evolution. The results of this work will support future studies on the *F. suspensa* mitochondrial genome, including comparative analyses of the *F. suspensa* organellar and nuclear genomes and studies on the evolutionary history and phylogenetic relationships of *F. suspensa*.

## Materials and methods

### Plant materials and genetic sequencing

Biennial branch cuttings of short-style *F. suspensa* grown at the botanical garden of Shanxi Agricultural University, Shanxi Province, China, were collected and planted in a shaded, 25 °C environment. After two weeks, the yellowed leaves were collected, sterilized with 75% alcohol, quick-frozen in liquid nitrogen, and stored at -80 °C.

The leaf materials of *F. suspensa* were sent to Genepioneer Biotechnologies (Nanjing, China) for sequencing. Total genomic DNA was extracted using the plant genomic DNA kit (Tiangen Biotech, Beijing, China). To obtain the full-length mitochondrial genome with high accuracy, short-read and long-read sequencing technologies were combined in this study. The short-read sequencing platform was Illumina Novaseq 6000 (Illumina, San Diego, CA, USA) and the paired end sequencing (PE) read length was 150 bp, Using the fastp (v0.20.0, https://github.com/OpenGene/fastp) software to filter the original data and get high-quality reads. The long-read sequencing platform was Nanopore PromethION (Nanopore, Oxford, UK), then the sequencing data was filtered by filtlong (v0.2.1, https://link.zhihu.com/?target=https%3 A//github.com/rrwick/Filtlong) software.

### Mitochondrial genome assembly

To assemble the *F. suspensa* mitochondrial genome, the comparison software Minimap2 (v2.1) [45] was used to compare the original long-read sequncing data with the reference gene sequence (plant mitochondrial core gene, https://github.com/xul962464/plant_mt_ref_gene), the sequence with similar fragment longer than 50 bp were selected as candidate sequences. The candidate sequences with more aligned genes (one sequence contains multiple core genes) and higher alignment quality (covering more complete core genes) were selected as the seed sequence. Then compared the original long-read sequencing data with the seed sequence, the sequences with minimum overlap of 1 kb and at least 70% similarity were added to seed sequence, and iteratively align the original data to the seed sequence, so as to obtain all long-read sequencing data of the mitochondrial genome. Finally, the assembly software canu [46] was used to correct the long-read sequencing data obtained, and bowtie2 (v2.3.5.1) was used to align the short-read sequencing data to the corrected sequence. Then, the default parameter Unicycler (v0.4.8) was used to compare the above short-read sequencing data and the corrected long-read sequencing data for concatenation. Finally, the *F. suspensa* mitochondrial genome was obtained.

### Mitochondrial genome structure annotation

The encoding protein and rRNA used BLAST to align the published plant mitochondrial sequences as refs; further manual adjustments are made for related species. tRNA was annotated using tRNAscan-SE (http://lowelab.ucsc.edu/tRNAscan-SE/) [47] and Open Reading Frame Finder (http://www.ncbi.nlm.nih.gov/gorf/gorf.html) was used to annotate ORF, the shortest length was set to 102 bp, and redundant sequences and sequences with overlap with known genes were excluded. Sequence alignments greater than 300 bp are annotated to the NR library. To obtain more accurate annotation results, the above results were checked and manually corrected. Finally, the *F. suspensa* mitochondrial genome was mapped using OGDRAW (https://chlorobox.mpimp-golm.mpg.de/OGDraw.html).

### Prediction of RNA editing sites

The Predictive RNA Editor for Plants (PREP) suite (http://prep.unl.edu/) was used to predict RNA editing sites in the mitochondrial genome. The PREP suite was also used to predict changes in the hydrophilic and hydrophobic properties of amino acids due to codon changes after RNA editing.

### Analysis of relative synonymous codon usage (RSCU)

The numerical value of RSCU is the ratio of the actual frequency of codon usage to the theoretical frequency of codon usage. A script wrote in Perl was used to filter Uniq CDS(choose one of multiple copies of CDS) and do the calculations.

### Analysis of repeated sequences

The repeat sequences include simple sequence repeats (SSRs), tandem sequence repeats (TSRs), and dispersed sequence repeats (DSRs). The MIcroSAtellite (MISA) identification tool Perl script was used to detect simple sequence repeats (v1.0, parameter:1–10 2–5 3–4 4 − 3 5 − 3 6 − 3). Tandem repeats (> 6 bp repeat units) were detected using Tandem Repeats Finder v4.09 software (http://tandem.bu.edu/trf/trf.submit.options.html) (trf409.linux64, parameter:2 7 7 80 10 50 2000 -f -d -m) with default parameters. Dispersed repeats were detected using blastn(v2.10.1, parameters: -word size 7, e-value 1e-5, remove redundancy, remove tandem duplication). Using circos v0.69-5 to visualize these repeats.

### Homologous sequence analyses of organellar and nuclear genomes

Homologous sequences between organellar and nuclear genomes were analyzed using BLAST software, the following screening criteria: matching rate 70%, e-value 1e -5, and length 30 bp. Homologous sequences were mapped using CIRCOS (v0.69-5). The chloroplast genome (NC_036367.1) and the nuclear genome (SRR10064062-SRR10064365) of *F. suspensa* were downloaded from NCBI database (https://www.ncbi.nlm.nih.gov/) as the comparison genome.

### Analyses of Ka/Ks and Pi

To understand the natural selection pressure in the evolution of the genus forsythia, homologous protein sequences between *F. suspensa* and the previously-published mitochondrial genomes of *O. europaea* (LR743801.1), *N. tabacum* (NC_006581.1), *Z. mays* (NC_007982.1), *G. max* (NC_020455.1) were obtained using BLASTN. Then, the shared protein-coding genes were aligned using MAFFT v7 (https://mafft.cbrc.jp/alignment/software/). The non-synonymous (Ka) and synonymous (Ks) ratios (Ka/Ks) were calculated using KaKs_Calculator v 2.0 (https://sourceforge.net/projects/kakscalculator2/) based on the MLWL method.

The homologous gene sequences of *F. suspensa* were globally aligned using MAFFT(v7.427, --auto), and the nucleotide diversity (Pi) value of each gene was calculated using dnasp5.

### Comparative and syntenic analyses of genome structures

We compared the mitochondrial genome structure of several related plant species with CGVIEW (http://stothard.afns.ualberta.ca/cgview_server/), using default parameters. Two approaches were used for the syntenic analyses of *F. suspensa* and four of its close relatives. First, NUCMER (4.0.0beta2) was used to align and compare the assembled genomic sequences and to generate the dot plot, using maxmatch parameters. Second, BLASTN (2.10.1+) software was used to draw collinearity plots, word size was set to 7, e-value was set to 1e-5, and fragments larger than 300 bp in length were screened and compared.

### Phylogenetic analysis

Since few plant mitochondrial genome data have been published, we selected the mitochondrial genome data of the model species and the closely related species of *F. suspensa*, as the NCBI database for phylogenetic analysis. The published mitochondrial genomes of 21 species were used to construct the *F. suspensa* phylogenic tree (Supplementary Table 2). The analysis included six Cruciferae species, four Gramineae species, three Chenopodiaceae species, two species each of Fabaceae and Salicaceae, and one species each from Magnoliaceae, Solanaceae, Ginkgoaceae, and Oleaceae. The CDS were extracted from the mitochondrial genomes of the 21 species and subjected to multiple sequence alignment using MAFFT v7.427. Maximum likelihood (ML) phylogenetic tree was conducted by RAxML v8.2.10 (https://cme.h-its.org/exelixis/software.html) (GTRGAMMA model) estimation with 1000 bootstrap replications. Bayesian inference (BI): The optimal nucleotide substitution model was calculated by jModelTest v2.1.10 (https://github.com/ddarriba/jmodeltest2), and then MrBayes v3.2.7a (http://nbisweden.github.io/MrBayes/) was used to establish Bayesian inference (BI) phylogenetic tree,the parameters of MrBayes v3.2.7 software are based on jModelTest v2.1.10 results.

### Electronic supplementary material

Below is the link to the electronic supplementary material.

Supplementary Material 1

## Data Availability

The sequence and annotation of *Forsythia suspensa* mt genome was submitted to the NCBI. The accession number in Gene Banks is OQ534870.

## Abbreviations

*F. suspensa*: *Forsythia suspensa* (Thunb.) Vahl
PCGs: Protein-coding genes
ORF: Open Reading Frame
RSCU: Relative synonymous codon usage
SSR: Simple sequence repeat
TSR: Tandem sequence repeat
DSR: Dispersed sequence repeat
Pi: Nucleic acid diversity
mtDNA: Mitochondrial DNA
gDNA: Genomic DNA
BLAST: Basic Local Alignment Search Tool
nr library: Nuclear library
NCBI: National Center for Biotechnology Information
CDS: Coding sequence
