# Supplementary material for: Assembly and analysis of the complete mitochondrial genome of Forsythia suspensa (Thunb.) Vahl
Source: BMC Genomics. 2023 Nov 23;24:708. doi: 10.1186/s12864-023-09821-4 (PMC10666317; doi:10.1186/s12864-023-09821-4)
Supplement: Supplementary file 1 — Supplementary Material 1 [file 12864_2023_9821_MOESM1_ESM.docx]

**Supplementary files**

**Table**

**Supplementary Table 1 Distribution of tandem repeats in the forsythia mitochondrial genome**

| NO. | Copy | Repeat sequence | Size | Percent Matches | Start | End |
| --- | --- | --- | --- | --- | --- | --- |
| 1 | 1.9 | ACTTCCGAAGATAAGAAGAAG | 22 | 85 | 44322 | 44362 |
| 2 | 2 | GTCGGAGCAGAACTACTATTAT | 22 | 91 | 56334 | 56377 |
| 3 | 1.9 | ACAAAGAAAGCCAAAG | 16 | 93 | 189448 | 189478 |
| 4 | 4.8 | TATTGATGATAGTACAA | 18 | 89 | 238018 | 238099 |
| 5 | 4.2 | ATATTGATGATAGTGACG | 18 | 94 | 238032 | 238107 |
| 6 | 7 | ATATTGATG | 9 | 70 | 238032 | 238094 |
| 7 | 2.6 | TATTGATGATAGTGACAATATTGATGATAGTGACGA | 36 | 89 | 238018 | 238107 |
| 8 | 23.5 | TA | 2 | 82 | 285457 | 285500 |
| 9 | 2.1 | TACTATATTAAACTAT | 16 | 100 | 364342 | 364375 |

**Supplementary** **Table 2 NCBI accession numbers for the mitochondrial genomes of the 21 species**

| No. | species | accession number |
| --- | --- | --- |
| 1 | *Beta vulgaris* subsp. *vulgaris* | BA000009.3 |
| 2 | *Magnolia biondii* | NC_049134.1 |
| 3 | *Brassica oleracea* | JF920286.1 |
| 4 | *Olea europaea* subsp. *europaea* | LR743801.1 |
| 5 | *Beta vulgaris* subsp. *vulgaris* | NC_002511.2 |
| 6 | *Nicotiana tabacum* | NC_006581.1 |
| 7 | *Zea mays* subsp. *mays* | NC_007982.1 |
| 8 | *Brassica napus* | NC_008285.1 |
| 9 | *Sorghum bicolor* | NC_008360.1 |
| 10 | *Oryza sativa Japonica Group* | NC_011033.1 |
| 11 | *Ginkgo biloba* | NC_027976.1 |
| 12 | *Brassica juncea* | NC_016123.1 |
| 13 | *Brassica rapa* subsp. *oleifera* | NC_016125.1 |
| 14 | *Salix purpurea* | NC_029693.1 |
| 15 | *Raphanus sativus* | NC_018551.1 |
| 16 | *Glycine max* | NC_020455.1 |
| 17 | *Styphnolobium japonicum* | NC_039596.1 |
| 18 | *Triticum aestivum* | NC_036024.1 |
| 19 | *Populus davidiana* | NC_035157.1 |
| 20 | *Chenopodium quinoa* | NC_041093.1 |
| 21 | *Capsella rubella* | NC_042883.1 |

**Figure**


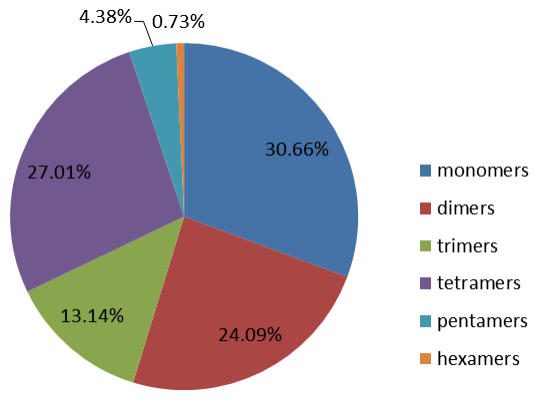


**Supplementary Figure 1 Simple repeat sequence statistics**


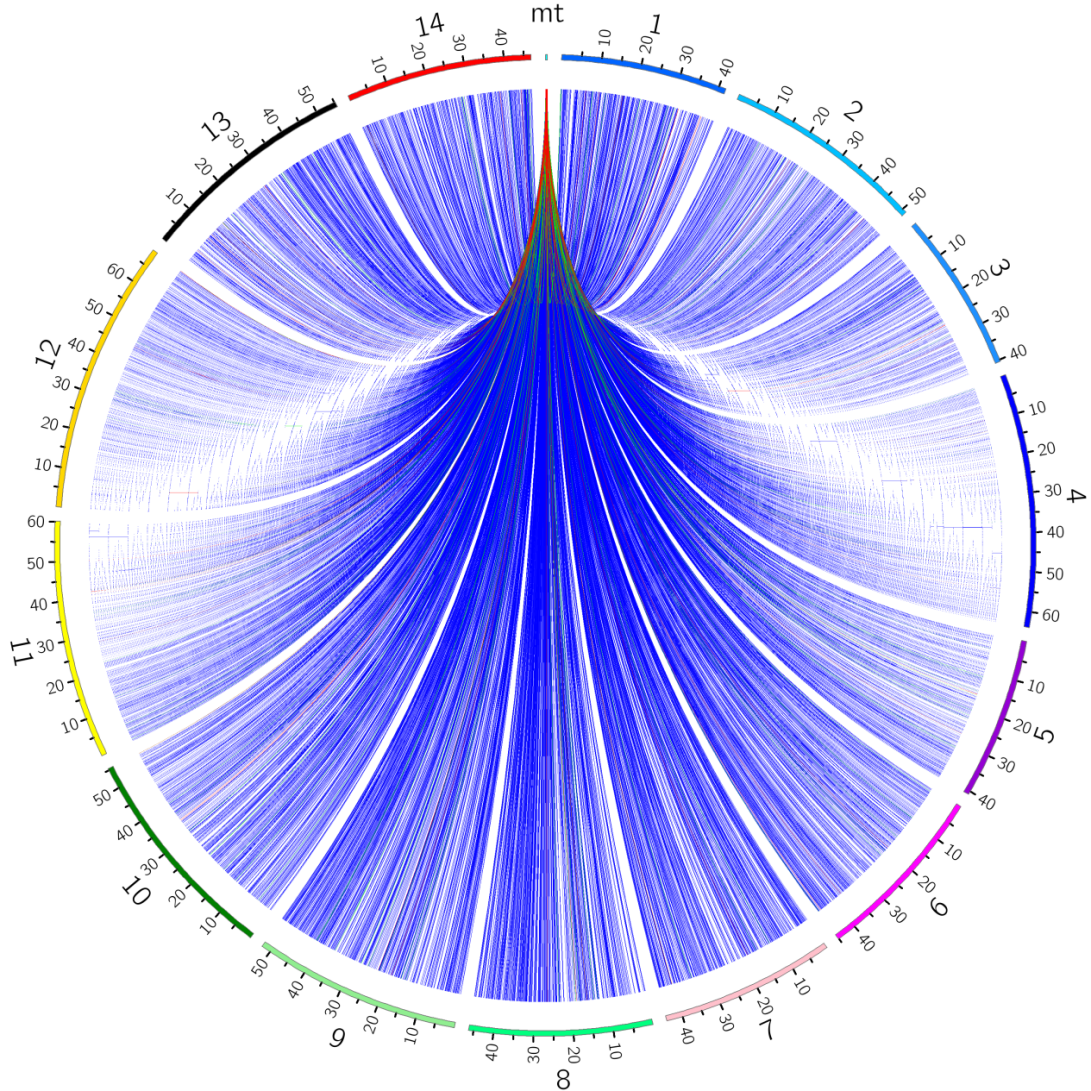


**Supplementary Figure 2 Alignment of *F.suspensa* mitochondrial genome to the nuclear genome**


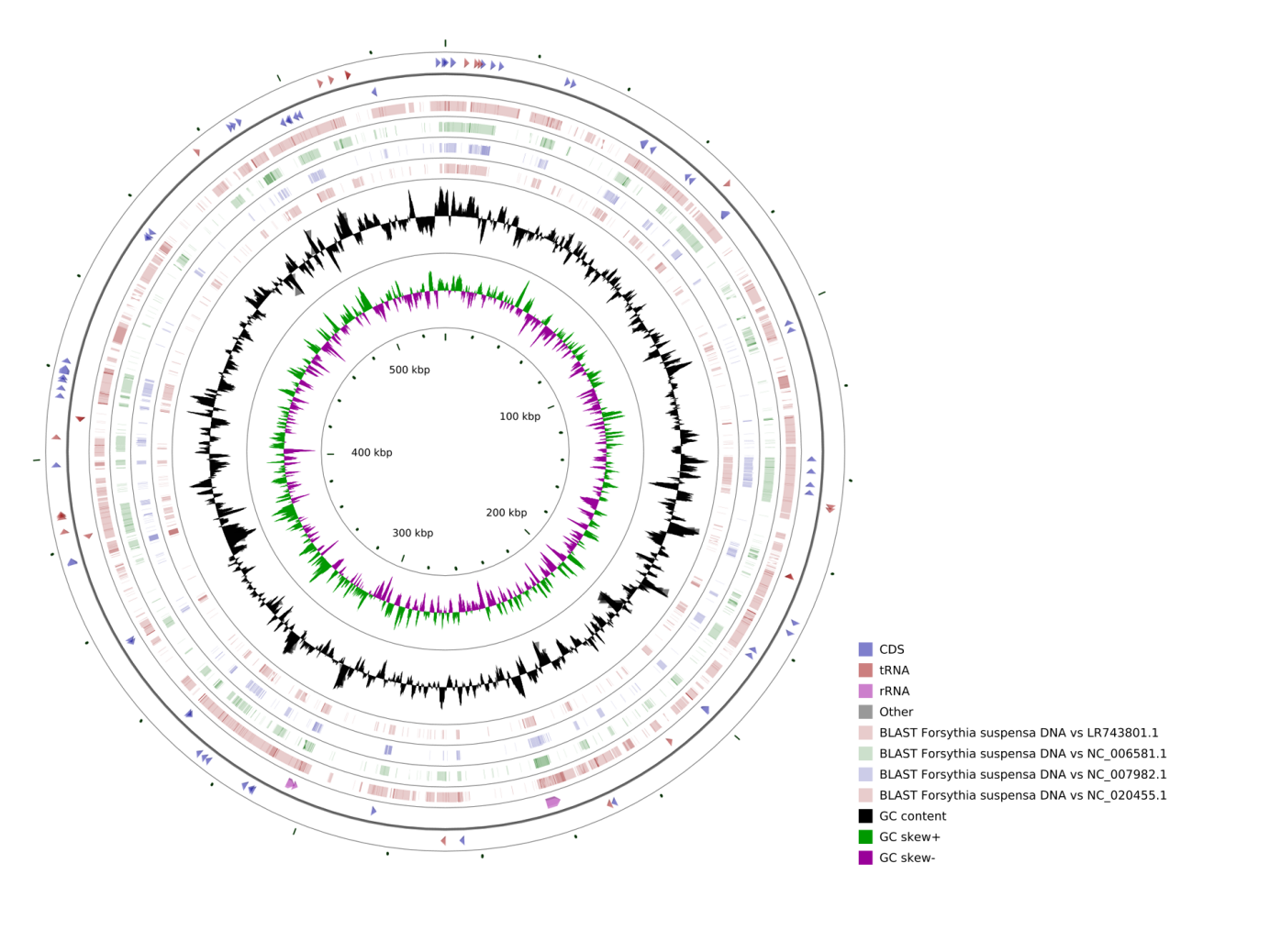


**Supplementary Figure 3 Comparative analysis of the mitochondrial structure of *F.suspensa***

Note: The top two most circles in the figure describe the gene length and direction of the genome; the inside circles represent the similarity results of the alignment with other reference genomes, the darker the higher the homology; the black circles represent the GC content.
